# Non-coding RNAs in the viral host-pathogen interaction: molecular regulation and therapeutic potential

**DOI:** 10.3389/fcimb.2025.1734182

**Published:** 2025-12-18

**Authors:** Maham Yamin, Nirmin Alsahafi, Rwaa Hussin Abdulal, Muhammad Asad, Mohammad Bosaeed, Ali Zohaib

**Affiliations:** 1 Rawalpindi Medical University, Rawalpindi, Pakistan; 2 Infectious Disease Research Department, King Abdullah International Medical Research Center, Jeddah, Saudi Arabia; 3 King Saud bin Abdulaziz University for Health Sciences, Jeddah, Saudi Arabia; 4 Ministry of the National Guard - Health Affairs, Jeddah, Saudi Arabia; 5 Huazhong Agricultural University, Wuhan, China; 6 Infectious Disease Research Department, King Abdullah International Medical Research Center, Riyadh, Saudi Arabia; 7 King Saud bin Abdulaziz University for Health Sciences, Riyadh, Saudi Arabia; 8 Ministry of the National Guard - Health Affairs, Riyadh, Saudi Arabia

**Keywords:** circRNA, host–pathogen interaction, long non-coding RNAs, microRNAs, non-coding RNAs, RNA therapeutics, viral infection

## Abstract

Non-coding RNAs (ncRNAs), including microRNA (miRNA), long non-coding RNA (lncRNA) and circular RNA (circRNA), serve as key regulatory molecules in the context of viral infection. They play dual roles by modulating host immune responses and influencing viral replication, persistence, and disease progression. Numerous ncRNAs have been implicated in infections caused by viruses such as HCV, DENV and SARS-CoV. This review highlights the biogenesis and multifaceted functions of both host-encoded and virus-encoded ncRNAs in shaping host-pathogen interactions. It also examines their potential as novel biomarkers and therapeutic agents for viral infections. We discuss translational applications such as Miravirsen, a miRNA inhibitor that reached clinical trials for Hepatitis C Virus (HCV) and diagnostic relevance of lncRNA NEAT1 in SARS-CoV-2 infection. In the end, we have also addressed the current challenges and limitations involved in translating research observations of ncRNAs to clinical outcomes.

## 1 Introduction

Emergence and reemergence of viral infections, such as SARS-CoV, MERS-CoV, Zika virus (ZIKV), Ebola virus, SARS-CoV-2, Crimean-Congo hemorrhagic fever virus (CCHFV) and influenza A virus (IAV), have persistently threatened global health (Schwartz, 2021). Moreover, factors such as climate change, global trade and an increase in international travel can contribute to the rapid spread of infectious diseases (Hoffman and Maldonado, 2024; Almulhim et al., 2025). In today’s globalized world, a small outbreak in one region can result in a global pandemic. In addition to these factors, these viral agents can rapidly evolve and evade the host’s immune surveillance system. The emergence of viral mutations at the genomic level is a persistent challenge and it continuously threatens public health. These mutations contribute towards virus escape, while on the other hand, they compromise vaccine effectiveness as well. In addition to this, these mutations can also result in false negative results, hindering effective surveillance efforts. This necessitates the development of novel diagnostic and therapeutic strategies (Han et al., 2023; Yu et al., 2024).

During viral infection, host immune system is activated and results in complex interactions. Although protein-based host-virus interactions have been extensively studied, little is known about the role of non-coding RNAs (ncRNAs) during viral infection. Recent studies have reported the integral role of ncRNAs, such as microRNA(miRNA), long non-coding RNAs (lncRNAs) and circular RNAs (circRNAs) during viral infection (Wang et al., 2017a; Liu et al., 2019a; Withers et al., 2019; Henzinger et al., 2020; Maarouf et al., 2023). One of the extensively studied ncRNAs in host-virus interactions is miRNA. miRNAs, either host-derived or virus-derived, have been reported to play a key role in virus infection and host immune responses. These small non-coding (sncRNA) RNAs target specific mRNAs involved in the host immune response mechanism and viral replication (Bannazadeh Baghi et al., 2024; Bahojb Mahdavi et al., 2025). Similarly, another class of ncRNAs, lncRNAs, has been extensively investigated during viral infections. Increasing evidence suggests that they regulate transcription, translation and protein interactions during viral infection (Liu et al., 2019a; Statello et al., 2021; Mattick et al., 2023). Recently, circRNAs have also been emerging as key regulators of immune responses during viral infection. circRNAs interact with miRNAs and modulate their interactions with target mRNAs, resulting in altered gene expression (Wang et al., 2017a; Belousova et al., 2018; Maarouf et al., 2023).

Given their essential roles in viral pathogenesis, ncRNAs are now increasingly explored for their potential as viral therapeutic or diagnostic tools. Several studies have reported that ncRNAs can inhibit viral infection. For example, miR-let-7c is a host-derived miRNA and has been reported to suppress the hepatitis C virus (HCV) replication (Chen et al., 2019). Similarly, lncRNA lncITPRIP-1 results in increased production of interferon, resulting in inhibition of HCV replication (Xie et al., 2018a). Like miRNAs and lncRNAs, circRNAs have been reported to inhibit viral replication via both direct and/or indirect mechanisms. For example, circRNA circVAMP3 not only directly inhibits IAV replication, but this specific circRNA also restores host immune responses against IAV (Min et al., 2023). Another miRNA, miR-122, is being studied as a promising therapeutic agent against HCV infection. Two antisense locked nucleic acid (LNA) inhibitors (i.e., Miravirsen and RG-101) have been developed. These LNA inhibitors directly target miR-122, which results in the effective suppression of HCV infection in chronic patients. However, following cessation of LNA inhibitor therapy, HCV rebound has been observed (Panigrahi et al., 2022).

Several studies have also reported the altered expression of ncRNAs during viral infection. Researchers are now focusing on the development of novel diagnostic and prognostic biomarkers using these altered expression profiles of ncRNAs during viral infections. Recently, Liu and colleagues have reported that COVID-19 patients display unique ncRNA expression patterns, correlating with disease severity (Liu et al., 2023). Another study on dengue virus (DENV) infection has reported that lncRNA NEAT1 displays altered expression patterns in dengue patients (Pandey et al., 2017). Taken together, these findings indicate that ncRNA expression patterns can be used as potential diagnostic and prognostic tools during viral infection.

## 2 Classification and biogenesis of ncRNAs

ncRNAs do not encode proteins, yet play a crucial role in gene expression. It is estimated that each cell has around 10 million RNA molecules, with ncRNAs constituting a significant portion of this total. Keeping in view their diverse functions, ncRNAs are divided into two groups: housekeeping ncRNAs and regulatory ncRNAs. Housekeeping RNAs include ribosomal RNA (rRNA), transcriptional RNA (tRNA) and small nuclear RNA (snRNA). These ncRNAs are generally involved in regulating cellular functions. Regulatory ncRNAs, on the other hand, are further subdivided based on their size. Those regulatory ncRNAs, which are longer than 200 nucleotides, are referred to as lncRNAs, whereas those shorter than 200 nucleotides are called smaller nuclear RNAs (snRNAs). The snRNAs further include miRNAs, small interfering RNA (siRNA) and piwi-interacting RNAs (piRNAs). circRNAs are another type of regulatory ncRNAs and are single stranded, covalently closed RNA molecules with sizes ranging from 100 nucleotides to 10,000 nucleotides (Belousova et al., 2018; Zhang et al., 2019; Caba et al., 2021; Cardon et al., 2021; Oo et al., 2022; Ghani et al., 2025).

**Figure 1:**
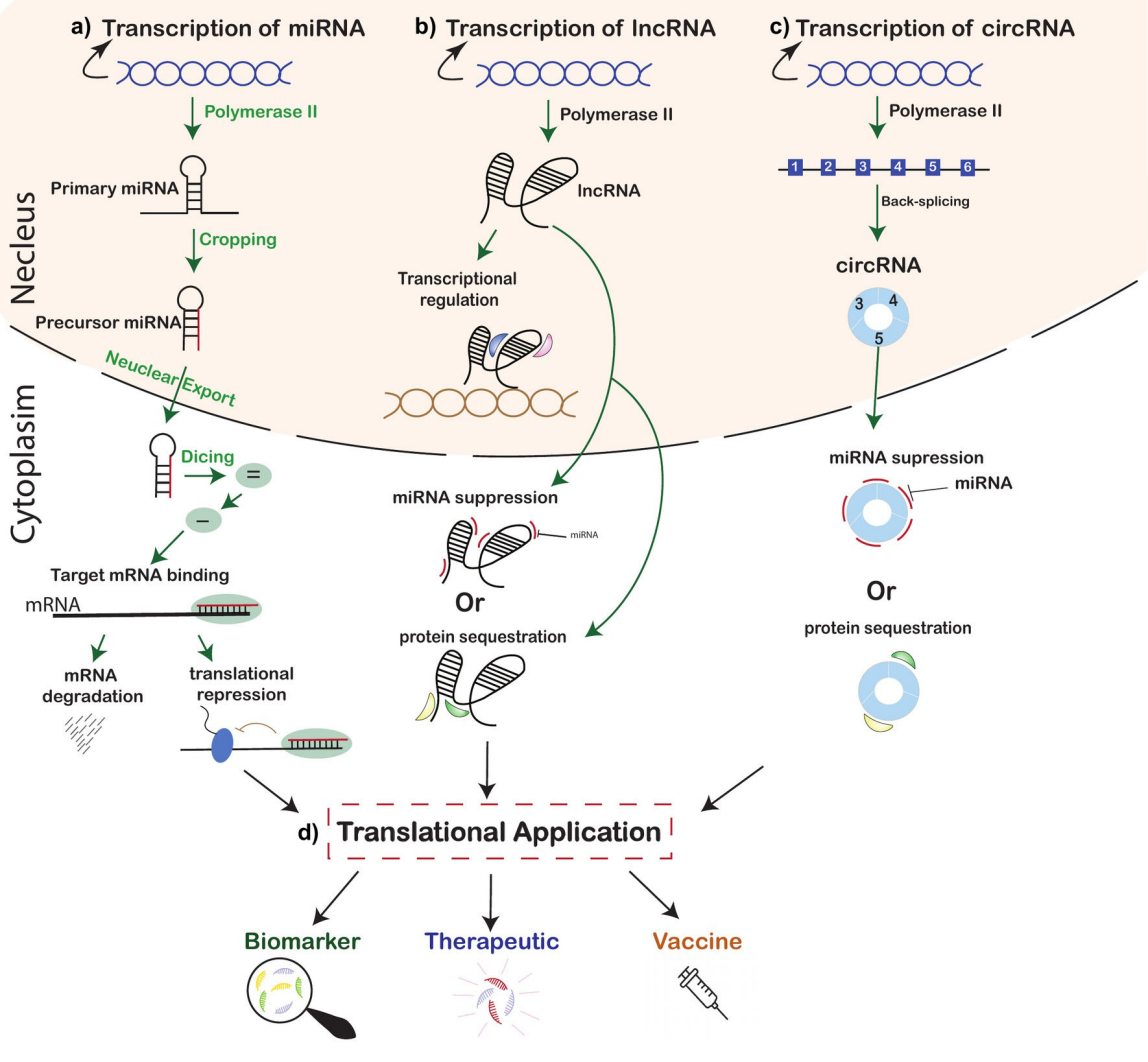
Biogenesis, mechanisms, and translational relevance of non-coding RNAs (ncRNAs). Schematic representation illustrating the major classes of non-coding RNAs and their biological and translational significance. **(a)** Biogenesis of microRNAs (miRNAs), transcribed by RNA polymerase II and sequentially processed through cropping and dicing to yield mature miRNAs that regulate gene expression via translational repression or mRNA degradation. **(b)** Biogenesis and functional roles of long non-coding RNAs (lncRNAs), transcribed by RNA polymerase II and acting at transcriptional and post-transcriptional levels as scaffolds, decoys, or modulators of miRNAs and proteins. **(c)** Formation and activity of circular RNAs (circRNAs), generated through back-splicing and functioning as molecular sponges or regulators of signaling pathways. **(d)** Translational applications: ncRNAs serve as promising diagnostic and prognostic biomarkers, therapeutic targets, and innovative vaccine platforms, bridging fundamental gene regulation with clinical translation.

Non-coding RNAs are produced through various biogenetic pathways involving various cellular machinery (**Figure 1**). miRNA biogenesis is a multi-step process that starts in the nucleus and concludes in cytoplasm. miRNAs are initially transcribed by RNA polymerase II and sometimes by polymerase III as long primary miRNAs (pri-miRNAs) transcripts. These pri-miRNAs possess characteristic stem-loop structures. Following transcription in the nucleus, pri-miRNAs are then processed by a microprocessor complex consisting of RNase III enzyme Drosha and its cofactor DGCR8. This processing results in precursor miRNAs (pre-miRNAs). Pre-miRNAs are typically around 70 nucleotides in length. These pre-miRNAs are then transported to the cytoplasm. Once in the cytoplasm, the pre-miRNAs are further processed by Dicer enzyme, which cleaves the pre-miRNAs to generate mature double-stranded miRNAs. One strand of this miRNA duplex is referred to as guide strand, is incorporated into the RNA-induced silencing complex (RISC). The second strand or passenger strand is usually broken down. The guide strand then directs RISC to target mRNAs to regulate gene expression (Balasubramaniam et al., 2018; O’Brien et al., 2018; Bannazadeh Baghi et al., 2024; Brillante et al., 2024; Hynes and Kakumani, 2024; Bahojb Mahdavi et al., 2025).

The lncRNAs are a diverse group of ncRNAs with varying biogenesis pathways. lncRNAs often share similarities with mRNA biogenesis but with some notable distinctions. The majority of lncRNAs are generated by RNA polymerase II, whereas some can be transcribed by polymerase I too. Although lncRNAs can undergo 5’ capping, splicing and 3’ polyadenylation like mRNAs, however; lncRNAs are often less efficiently spliced or polyadenylated. lncRNAs can originate from different genomic locations, including introns and exons of protein-coding genes. Interestingly, some lncRNAs can serve as a precursor for miRNAs (Marchese et al., 2017; Suarez et al., 2020; Statello et al., 2021; Nojima and Proudfoot, 2022; Mattick et al., 2023).

circRNAs are endogenous ncRNAs that lack 5’ caps and 3’ poly(A) ends and have a covalently closed loop structure, which is formed through a unique splicing event. circRNAs are primarily produced by an alternative splicing mechanism called back-splicing or head-to-tail splicing (Belousova et al., 2018; Caba et al., 2021; Hwang and Kim, 2024). This occurs when a downstream 5’ splice donor site combines with an upstream 3’ splice acceptor site (Kristensen et al., 2019). This non-canonical splicing is enabled by complementary inverted repeat sequences, such as Alu elements or RNA-binding proteins (RBPs), which bring splice sites nearby. Depending on the sequences they comprise, there are various varieties of circRNAs (Ivanov et al., 2015). Exonic circRNAs are composed exclusively of exons. Circular intronic RNAs are produced from intronic lariats that do not undergo debranching. Intronic and exonic sequences are maintained by exon-intron circRNAs. Some factors that rigorously regulate the synthesis of circRNAs by either promoting or stopping circularization include MBL/MBNL1, QKI and FUS. circRNAs are generally more stable than linear RNAs due to their exonuclease resistance. Most circular circRNAs are in the cytoplasm; however, specific nuclear circRNAs have also been identified (Maass et al., 2017).

## 3 The role of virus-produced ncRNAs in viral infection and pathogenesis

Virus-produced ncRNAs, such as miRNAs, lncRNAs and circRNAs, play essential roles during viral infection. By modulating the host immune responses, these ncRNAs promote viral infection and pathogenesis (Tycowski et al., 2015). For example, Epstein-Barr virus (EBV) produces a variety of viral miRNAs, including miR-BARTs. miR-BART-7 and miR-BART-9 can modulate virus life cycle and cell proliferation (Caetano et al., 2024). Several studies also report that miR-BARTs can target host immune-related genes to prevent apoptosis and to avoid immune surveillance. These miRNAs can also alter viral gene expression and contribute towards virus latency (Iizasa et al., 2020; Ungerleider et al., 2021). Similarly, the Kaposi’s sarcoma-associated herpesvirus (KSHV) produces miRNAs that increase the virus’s ability to proliferate within the host by inhibiting the host’s antiviral responses (Zhao et al., 2023). In case of human immunodeficiency virus (HIV), viral miRNAs can modulate cell signaling pathways to promote immune evasion and chronic infection (Balasubramaniam et al., 2018). Similarly, lncRNA PAN, which is also produced by KSHV, regulates the viral gene expression (Rossetto and Pari, 2014). Several other viral lncRNAs have been reported to modify host cellular pathways to enhance viral persistence in the host (Liu et al., 2019a).

Recently, several studies have reported that circRNAs produced by certain viruses play essential roles in viral pathogenicity. Research indicates that specific viral circRNAs may function as sponges for host miRNAs, affecting the host’s immune response and cellular milieu (Panda, 2018). The circRNAs can sequester miRNAs that typically inhibit viral replication or stimulate antiviral pathways, augmenting the virus’s capacity to replicate within host cells (Wang et al., 2024).

## 4 miRNAs as regulatory molecules in viral infections

### 4.1 Introduction to miRNAs

miRNAs are a class of small, non-coding RNA molecules typically 18–25 nucleotides in length that play essential roles in the post-transcriptional regulation of gene expression. miRNAs function by binding to complementary sequences in the 3′ untranslated regions (3′ UTRs) of target messenger RNAs (mRNAs). This results in the degradation or translational repression of the target mRNAs (O’Brien et al., 2018; Bahojb Mahdavi et al., 2025).

### 4.2 miRNAs in viral replication and host signaling regulation

miRNAs not only regulate cellular signaling but, also play a critical role during viral replication (Bannazadeh Baghi et al., 2024; Bahojb Mahdavi et al., 2025). They affect viral replication by targeting either viral genome and/or by modulating the host immune responses (**Table 1**). Viral miRNAs aid in virus replication by targeting genes involved in host immune response mechanisms (Liao et al., 2021b; Bannazadeh Baghi et al., 2024; Bahojb Mahdavi et al., 2025). Conversely, the host-derived miRNAs can directly suppress viral RNAs or modulate host response mechanisms. For example, miR-22 has been reported to promote HCV replication by binding to 5UTR of HCV (Kunden et al., 2020). Similarly, miR-210 and miR-199a-3p have been reported to inhibit Hepatitis B Virus (HBV) (Zhang et al., 2010). During Japanese encephalitis virus (JEV) infection, expression of RNF11 is regulated by miR-19b-3p, which results in modulation of antiviral immune responses (Zhu et al., 2015). Recently, miR-98-5p has been identified as a potential suppressor of SARS-CoV-2 replication (Rizkita and Astuti, 2021). On the other hand, miR-146a modulates TRAF6 and promotes IAV replication (Yan et al., 2025). Similarly, miR-373 facilitates viral replication during HSV-1 infection (Dass et al., 2023). miRNA-mediated regulation of neuroinflammation has also been reported during HSV-1 infection (Xie et al., 2018b; Patrycy et al., 2022). During ZIKV infection, miRNAs also modulate host immune responses and fetal development (Carvalho-Silva et al., 2022; Lai et al., 2025). DENV is another arbovirus like ZIKV, which interacts with host miRNAs to regulate host-pathogen interactions. For instance, miR-4488 contributes to cellular immune responses during DENV infection and may serve as a potential biomarker (Filip et al., 2025).

**Table 1:** Overview of miRNAs experimentally shown to regulate viral replication of human pathogenic viruses. The table presents their described effects, associated virus and reference sources.

miRNA	Role in viral replication	Associated virus	Reference
miR-122	Pro-viral effect for hepatitis C virus (HCV) replication, Anti-viral effect in case of Hepatitis B Virus (HBV)	HCV, HBV	(Chen et al., 2011; Kunden et al., 2020)
miR-1 miR-15b miR-372/373 miR-501	Promotes HBV replication	HBV	(Guo et al., 2011; Jin et al., 2013; Zhang et al., 2013; Dai et al., 2014)
miR-501-3p miR-619-3p	Promotes HCV replication	HCV	(Herzog et al., 2020)
miR-27a miR-29 miR-130a miR-181c miR-182 miR-194 miR-196 miR-199a-3p miR-200c miR-296 miR-351 miR-431 miR-448 miR-548m let-7b	Inhibits HCV replication	HCV	(Hou et al., 2010; Hoffmann et al., 2012; Choi et al., 2014; Li et al., 2014, Li et al., 2022; Mukherjee et al., 2014; Wang and Li, 2018)
miR-28 miR-125b miR-150 miR-223 miR-382	Contributes towards human immunodeficiency virus (HIV) latency	HIV	(Huang et al., 2007)
miR29a	Inhibit HIV-1 replication	HIV-1	(Ahluwalia et al., 2008)
miR-127-3p miR-323 miR-486-5p miR-491 miR-387b-5p miR-593-5p miR-654	Inhibit influenza A virus (IAV) replication	IAV	(Song et al., 2010; Peng et al., 2018; Szczesniak et al., 2023)
miRNA-133a miR-548g-3p miR-484 miR-744	Inhibits dengue virus (DENV) replication	DENV	(Wen et al., 2015; Castillo et al., 2016; Castrillón-Betancur and Urcuqui-Inchima, 2017)
miR-36 miR-1258 miR-1293	Inhibits Kaposi’s sarcoma-associated herpesvirus (KSHV) replication	KSHV	(Kang et al., 2011; Yan et al., 2014; Hussein and Akula, 2017)
miR-15a miR-98-5p miR-153 miR-185 miR-298 miR-508 miR-1909 miR-3130	Inhibits SARS-CoV-2 replication	SARS-CoV-2	(Matarese et al., 2020; Ahmed et al., 2023; Vaddadi et al., 2023)

miRNAs can modulate the host immune responses in diversified ways, from expression regulation of pro-inflammatory cytokines to influencing the activity of pattern recognition receptors (PPRs). For example, during viral infection, miR-155 is upregulated, resulting in enhanced interferon production and stronger TH1 responses (Arroyo et al., 2020). Interestingly, miR-146 plays a dual role during viral infection. It can behave as pro-viral or anti-viral, depending upon the context (Nahand et al., 2020). Recently, Chen and colleagues have reported that miR-155 disrupts the mitochondrial homeostasis and regulates interferon responses (Chen et al., 2023). Another miRNA, miR-29, regulates IFN-γ production and Th1 cell development by suppressing T-bet and Eomes (Steiner et al., 2011). Moreover, miR-150 and miR-223 have been reported to attenuate STAT1 signaling in T cells (Moles et al., 2015). Another study highlights that miR-126-5p negatively regulates host antiviral immune responses (Wang et al., 2022).

### 4.3 Translational applications of miRNAs

RNA-based therapeutics, for example, miRNA mimics and inhibitors, may offer unique opportunities to combat viral infections. They target either host immune responses or virus-specific molecular pathways. One such example is Miravirsen, which is the inaugural miRNA anti-HCV therapeutic. It functions by inhibiting miR-122, which is a proviral miRNA (Brillante et al., 2024). Research is being conducted to explore the potential of miRNA-based antiviral therapies. For instance, miR-122 mimics are being studied for their potential to suppress HBV replication (Mahmoudian-Sani et al., 2019). Additionally, circulating miR-122 has also emerged as a potential biomarker for liver infection, especially in case of HCV infection (Ullah et al., 2022). Similarly, miR-199A-3p is being investigated as a potential therapeutic for HBV. Additionally, anti-miR-210 is being researched to stop cancer-causing effects in liver diseases related to HBV (Zhang et al., 2010).

Studies have shown that viruses can alter host miRNA expression profiles and understanding these miRNA-virus interactions may offer a basis for miRNA-based diagnostic and therapeutic tools. For instance, altered miRNA expression profiles have been observed during SARS-CoV-2 infection. Some host-derived miRNAs were found to be specifically depleted during SARS-CoV-2 infection (Bartoszewski et al., 2020). On the contrary, certain miRNAs are upregulated and contribute towards inflammation and lung injury during viral infection. Targeting these host-virus-miRNAs interactions can result in novel therapeutics.

Circulating miRNAs are stable in serum and can also serve as diagnostic biomarkers. During DENV infection, miR-150 expression levels have been reported to correlate with disease severity (Chen et al., 2014). Wong and colleagues have recently reported that miR-21-5p, miR-146a-5p, miR-590-5p, miR-188-5p and miR-152-3p can be used to detect DENV infection (Wong et al., 2020). Furthermore, miRNA profile in correlation with COVID-19 disease severity has also been reported (Pius-Sadowska et al., 2025). Collectively, these findings indicate the promising potential of miRNA-based therapeutics and diagnostics as new approaches against viral infections.

## 5 lncRNAs as regulatory molecules in viral infections

### 5.1 Introduction to lncRNAs

lncRNAs are RNA molecules longer than two hundred nucleotides. They do not code for proteins; however, they play important roles in gene regulation at transcriptional and post-transcriptional levels. Increasing evidence suggests that lncRNAs play important roles during viral infection (Liu et al., 2019a; Statello et al., 2021). Certain lncRNAs enhance viral replication or alter the host cell environment to facilitate viral pathogenesis, whereas others may alter interferon signaling to block infection and support viral clearance. Their wide range of functions indicates that lncRNAs can serve as important candidates for diagnostic and therapeutic applications (Liu et al., 2019a; Aldweik et al., 2025).

### 5.2 lncRNAs in viral replication and host signaling regulation

lncRNAs have recently been identified as key regulators of the innate immune system and cellular defenses against viral infections. Rather than encoding proteins, these molecules function as modulators that influence key immune signaling pathways, including NF-κB, IRF3 and JAK/STAT. By interacting with transcription factors, chromatin modifiers and RNA-binding proteins, lncRNAs determine whether viral infections are effectively recognized and eliminated or allowed to persist in the host (**Table 2**). For example, interferon-inducible lncRNAs such as USP30-AS1, NRAV, TSPOAP1-AS1, lnc-MxA and lnc-Lsm3b play pivotal roles in fine-tuning interferon signaling and host antiviral immune responses during IAV infection (Wang and Cen, 2020; Wang et al., 2022; Cao et al., 2025). Similarly, IVRPIE enhances host defense against the influenza A virus by upregulating IFN-β1 and key ISGs through histone modification (Zhao et al., 2020). Another lncRNA#32 promotes type I IFN signaling and ISG expression, thereby restricting EMCV, HBV and HCV replication (Nishitsuji et al., 2016). lncRNA IFITM4P stabilizes IFITM1/2/3 expression by sponging miR-24-3p, which results in increased innate antiviral responses (Xiao et al., 2021).

**Table 2:** Overview of lncRNAs experimentally shown to regulate viral replication of human pathogenic viruses. The table presents their described effects, associated virus and reference sources.

lncRNA	Role in viral replication	Associated virus	Reference
lncRNA Dreh	Repress HBV‐related HCC growth	HBV	(Huang et al., 2013)
LncRNA SEMA6A‐As1	Low expression is associated with poor prognosis in HBV‐related HCC.	HBV	(Yu et al., 2020b)
lncRNA NRON	Suppress viral transcription	HIV	(Li et al., 2016)
lncRNA#32	Regulates encephalomyocarditis virus (EMCV), HBV and HCV replication	EMCV, HBV, HCV	(Nishitsuji et al., 2016)
lncRNA-IFI6	Regulates antiviral innate immunity	HCV	(Liu et al., 2019b)
lncRNA MALAT1	A promoter of HIV-1 transcription	HIV	(Qu et al., 2019)
lncRNA NRAV	Modulates antiviral immune responses	IAV	(Ouyang et al., 2014)
lncRNA TSPOAP1	Promotes IAV replication	IAV	(Wang et al., 2022)
lncRNA NRAV	Promotes RSV replication	RSV	(Li et al., 2020a)
lncRNA BC200	Promotes Epstein-Barr virus (EBV) associated nasopharyngeal carcinoma	EBV	(Zhang et al., 2024)
lncRNA FANCI-2	Play role in modulating oncogenic pathways in human papillomavirus (HPV) related cancers	HPV	(Liu et al., 2025)
lncRNA U90926	Promotes herpes simplex virus (HSV) proliferation	HSV	(Shirahama et al., 2020)
lncRNA PIRAT lncRNA LUCAT1	Host immune regulation during SARS-CoV-2 infection	SARS-CoV-2	(Aznaourova et al., 2022)
lncRNA VILMIR	Host immune regulation during SARS-CoV-2 and RSV infections	SARS-CoV-2, RSV	(John et al., 2025)
lncRNA CHROMR	Regulates host antiviral immune responses	SARS-CoV-2 IAV	(Van Solingen et al., 2022)
lncRNA ANRIL lncRNA THRIL lncRNA NEAT1	Potential circulatory biomarkers	SARS-CoV-2	(Rahni et al., 2023)

Numerous lncRNAs have been documented to enhance viral propagation by inhibiting host immune responses. For instance, lncRNA-BTX enhances viral replication by modulating the localization of RNA-binding proteins DHX9 and ILF3 (Cao et al., 2023). Recently, Yan and colleagues have reported that lncRNA-DRNR disrupts the STAT1 activation. This disruption results in the reduced IFN-β mediated antiviral responses (Yan et al., 2024). Another lncRNA, THRIL, has been reported to inhibit IRF3 activation. This inhibition results in a reduction in type I and III interferons and enhanced IAV replication (Chen et al., 2025).

Moreover, several studies have also reported the integral roles of lncRNAs in various host signaling pathways. For example, lncRNA-ACOD1 interacts with GOT2 enzyme, which facilitates viral replication (Wang et al., 2017b). Another lncRNA, HEAL, interacts with FUS protein during HIV infection and facilitates HIV transcription (Chao et al., 2019). A recent study suggests that EBV-BART lncRNAs regulate the host gene expression (Verhoeven et al., 2019).

### 5.3 Translational applications of lncRNAs

Many lncRNAs have been reported to display expression patterns that correlate with the stage and often severity of viral infection. In recent studies, specific lncRNA expression patterns were observed during influenza and SARS-CoV-2 infections, which can aid in early diagnosis of these viral infections (Van Solingen et al., 2022; Aldweik et al., 2025). High-throughput sequencing has also revealed several differentially expressed lncRNAs during viral infections. In a study, a group of seven lncRNAs has been developed to effectively differentiate between severe and non-severe cases of COVID-19 (Cheng et al., 2021). Another study has also reported that lncRNAs such as HOTAIRM1, PVT1 and AL392172.1 are important regulators of SARS-CoV-2 infection (Moazzam-Jazi et al., 2021). Furthermore, studies conducted with EV71 and DENV have also reported that lncRNAs are associated with immune responses (Wang et al., 2017c; Li et al., 2018). Similarly, during ZIKV infection, altered expression of lncRNAs in neuroprogenitor cells has also been observed (Venkatesan et al., 2022). Recently, Arslan and colleagues have reported that lncRNAs such as ERCP FER1L4 and LOC1100133669 can be used as potential biomarkers to determine the disease severity during CCHFV-mediated infection (Arslan et al., 2022). Taken together, these findings indicate that lncRNAs can be used as novel tools not only to identify viral infection, but also to assess the severity of the disease. However, developing a standardized method to detect and quantify lncRNAs is an open venue, requiring further research.

Similar to diagnostic, therapeutic potential of lncRNAs is also being studied. Several studies have reported both pro- and anti-viral lncRNAs. For example, lncRNA-ACOD1 has been reported to be upregulated during several viral infections, indicating its potential pro-viral activity (Wang et al., 2017b). Moreover, research is being carried out to target virus encoded lncRNAs to determine their use in antiviral therapies. For instance, EBV sisRNAs or KSHV PAN RNA have been targeted to assess the impacts on viral latency and immune clearance (Min et al., 2022; Media et al., 2025).

Moreover, altering certain host-derived lncRNAs expression profiles can aid in the reversal of virus latency and possible approach for eradicating chronic infections (Kulkarni et al., 2023). Current research is focused on optimizing delivery mechanisms, such as lipid nanoparticles and plant-derived exosomes, for the administration of synthetic lncRNAs or CRISPR modulators (Kazemian et al., 2022; Hillman, 2023).

## 6 circRNAs as regulatory molecules in viral infections

### 6.1 Introduction to circRNAs

circRNAs are noncoding RNA without a 5′-cap structure or 3′ poly(A) tail. Their 3′ and 5′ ends are covalently bonded, forming a closed circular shape. circRNAs are generated from viral genome transcription/splicing or from host cell gene splicing. These ncRNAs play an important role in regulating host immune responses and viral replication (Belousova et al., 2018; Panda, 2018; Maarouf et al., 2023; Wang et al., 2024).

### 6.2 circRNAs in viral replication and host signaling regulation

Recent evidence shows that circRNAs can regulate viral replication by acting as competitive endogenous RNAs (ceRNAs) (**Table 3**). For example, in Hantaan virus infection, circ_0000479 was shown to sponge miR-149-5p, thereby lifting miR-149-5p suppression of RIG-I, which likely enhances antiviral defense (Lu et al., 2020; Yin et al., 2024). In another study with MERS-CoV, knockdown of host circRNAs (e.g., circFNDC3B, circCNOT1) led to significantly reduced viral load, consistent with a ceRNA-based regulatory mechanism (Zhang et al., 2020). Host circRNAs can act as miRNA sponges during viral infection, binding intracellular miRNAs to block their activity and modulate host gene expression, ultimately creating conditions favorable for viral replication. circRNAs–miRNAs interactions are vital in HBV-related hepatocellular carcinoma (HCC). Rui Liao and colleagues studied circRNA_101764, circRNA_100338, circ-ARL3 and circ-ATP5H in HBV-HCC, showing their roles in tumor development and metastasis (Liao et al., 2021a). circRNAs appear to play important roles in virus-induced immune responses. Li and colleagues have recently performed genome-wide sequencing of brain tissues from Japanese encephalitis virus (JEV)-infected mice. This study has revealed the altered expression of circRNA hsa_circ_0000220 (Li et al., 2020b). Which acts like a sponge for miR-326-3p, leading to an increase in cytokine production. Recent studies also indicate that disruptions in ceRNA networks, which involve lncRNAs, circRNAs and ncRNAs, play significant roles in the pathogenesis of SARS-CoV-2 (Aghajani Mir, 2024).

**Table 3:** Overview of circRNAs experimentally shown to regulate viral replication of human pathogenic viruses. The table presents their described effects, associated virus and reference sources.

circRNA	Viral infection	Mode of action	Reference
circ_0000479	Hantaan Virus	Indirectly regulated RIG-I expression by sponging miR-149-5p	(Lu et al., 2020)
circ_MerTK	IAV	Negatively regulates innate immune response	(Qiu et al., 2023)
circ_VAMP3	IAV	Restricts IAV replication	(Min et al., 2023)
circ_Slco3a1 circ_Wdr33	IAV	May be involved in IAV induced lung injury	(Wang et al., 2021)
circ_0000479	SARS-CoV-2	Regulates the immune response against SARS-CoV-2	(Firoozi et al., 2022)
circRNA_10156	HBV	Pro-tumorigenic	(Wang et al., 2020)
cFAM210A	HBV	Inhibits HCC tumorigenesis	(Yu et al., 2023)
circ_0000976, circ_0007750 and circ_013989	HBV-related HCC	Diagnostic panel for HCC	(Yu et al., 2020a)
circ_0001400	KSHV	Aids in maintaining latent infection	(Tagawa et al., 2023)
circ_ARFGEF	KSHV	Regulates KSHV mediated oncogenesis	(Yao et al., 2021)
circ_0007321	ZIKV	Regulates Zika virus replication	(Kang et al., 2023)
circ_FNDC3B circ_CNOT1	MERS-COV	Regulates virus replication	(Zhang et al., 2020)
circRNA_101764 circRNA_100338 circ-ARL3 circ-ATP5H	HBV-related HCC	Regulation of HCC associated tumorigenesis	(Liao et al., 2021a)
hsa_circ_0000220	Japanese Encephalitis Virus (JEV)	Regulates immune responses and inflammation	(Li et al., 2020b)

circRNAs can also alter essential cellular pathways by modulating protein stability, phosphorylation and ubiquitination. For instance, host-derived circ_0001400 binds to splicing factor PNISR and downregulates the KSHV gene expression. This results in aversion of apoptosis and development of latency (Tagawa et al., 2023). During IAV infection, the host-derived circVAMP3 disrupts the NP-polymerase interactions as circVAMP3 acts as a decoy for viral nucleoprotein (NP) and non-structural protein 1 (NS1). This interference restores interferon-β production and suppresses viral replication (Min et al., 2023). Together, these findings indicate that circRNA–protein interactions influence both viral pathogenesis and host immune responses. During EV-A71 infection, hsa_circ_0045431 binds hsa_miR-584, forming the circRNAs/NLRP3 axis that activates pyroptosis (Hu et al., 2023). Similarly, hsa_circ_0007321, derived from the DIS3L2 gene, has been reported to play an important role during ZIKV infection.

This circRNA, which is derived from the host, was shown to regulate the viral replication pathway by sponging miR-492. This, in turn, affects the protein NFKBID, which is a negative regulator of the NF-κB pathway (Kang et al., 2023).

In another interesting study, purified circRNAs were introduced into HeLa cells to observe their effect. They seemed to trigger a strong immune response, which was also demonstrated by increased expression of antiviral genes such as RIG-I, PKR, MDA5 and OAS1. As expected, this made the cells more resistant to viral infection and lower infection rates were observed in subsequent assays (Chen et al., 2017; Liu and Chen, 2022).

### 6.3 Translational applications of circRNAs

Due to their covalently closed structure, circRNAs are highly stable in serum samples, rendering them ideal candidates for the novel diagnostic tools. Moreover, as they are key regulators of immune responses against viral infections, they are being investigated for potential targets for therapeutic interventions. It has recently been reported that CRISPR techniques can be utilized to knock out circRNAs and their role inside different cellular pathways can be further studied (Santer et al., 2019; Yin et al., 2024). A recent study has also reported that circRNAs can be used to diagnose HBV infection (Jiang et al., 2020). Similarly, another circRNA, circ_3205, can aid in the COVID-19 diagnosis. Moreover, circ_3205 is a virus-encoded ncRNA, which also acts as a has-miR-298 sponge and positively regulates SARS-CoV-2 infection (Barbagallo et al., 2022).

Not only are circRNAs highly stable, but they also trigger strong immune responses, making them ideal candidates for RNA-based vaccine platforms. Recently, a study has investigated a potential circRNA vaccine encoding receptor binding domain (RBD) of SARS-CoV-2. This vaccine elicited robust T-cell responses, which may provide broad protection against different variants (e.g., Delta and Omicron) of SARS-CoV-2 (Qu et al., 2022). Amaya and colleagues have further explored the potential of circRNA vaccine in murine tumor models and their study revealed effective CD8+ T cell responses by dendritic cells (Amaya et al., 2023). Initially, scientists were struggling with the circularization techniques; however, with emerging research in synthetic biology, novel circularization techniques are being developed to synthesize circular RNA (Abe et al., 2018). Progress in molecular virology and host immunology, along with synthetic biology, has greatly improved our understanding of ncRNAs-mediated host-pathogen interactions, paving the way for improved diagnostics and therapeutics.

## 7 Challenges in non-coding RNA translational research for viral diagnosis, biomarkers and therapeutics

Despite their promising potential, the translational aspect of ncRNAs faces several challenges, which are also active areas of research. Due to significant variability among sample collection, type of samples, processing strategies and storage conditions, the ncRNA profiles obtained are highly variable. Low abundance of ncRNAs and sequence similarity are also major challenges, in addition to quantification techniques, which have all been originally developed for longer RNAs. All these factors make detecting small fold changes in ncRNAs during infections a major challenge.

Additionally, ncRNAs exhibit diverse forms and functions and only a small number have been identified and functionally characterized (Kulkarni et al., 2023). Some ncRNAs are present in normal pathophysiological states as well, such as inflammation and tissue injury and are not specific to a particular infection, which makes it difficult to rely on them as specific biomarkers of infection. For example, some lncRNAs (such as LINCO2574, GAPLINC) are involved in regulating innate antiviral immunity but, also show overlapping activation in unrelated infections (Rai et al., 2022; Chen et al., 2024).

One of the major challenges in using ncRNAs as therapeutic agents is their efficient and tissue specific delivery. Even though significant progress has been made with delivery systems as lipid nanoparticles and exosomes, poor penetration and limited organ specificity are still considered major hurdles in ncRNAs application as therapeutic agents. The ncRNAs-based therapeutics also risk off target gene modulation due to not only sequence complementarity, but also competition with endogenous RNAs. Due to all these limitations, very few ncRNAs have reached advanced clinical stages as therapeutic agents, despite promising laboratory results.

## 8 Future directions

ncRNAs have emerged as central regulators of viral pathogenesis and have also been shown to play an important role in shaping the immune response to viral infections. As fine-tuned regulators, they modulate pattern recognition receptors (PRRs) as well as downstream cytokine and chemokine networks to either activate or suppress antiviral pathways (Tycowski et al., 2015; Zhang et al., 2019; Suarez et al., 2020). These mechanisms regulate the balance between pro- and anti-inflammatory responses, which determine the severity of viral infections. Several ncRNAs directly target viral RNA and interfere with its translation or stability, while others influence the differentiation and activation of macrophages and dendritic cells. The noncoding RNAs can also modulate host epigenetic landscapes, resulting in altered gene expression profiles that may favor either viral persistence or clearance (Tycowski et al., 2015; Wang, 2018; Zhang et al., 2019; Suarez et al., 2020).

Early clinical experience with using ncRNAs as therapeutic and diagnostic agents has been encouraging to an extent. For example, in the case of Miravirsen, which is an LNA based anti-miR against liver enriched miR-122, a dose dependent reduction was observed in the HCV RNA in patients, which showed its clinical effectiveness (Janssen et al., 2013). Additionally, lncRNA NEAT1 has been consistently reported as being upregulated in SARS-CoV-2 positive samples, which could serve as a potential biomarker (Rodrigues et al., 2021). However, a study using miR-34 mimic, a potential cancer therapeutic, had to be halted in phase-1 clinical trial due to immune related issues (Hong et al., 2020).

Understanding specific functions of ncRNAs requires advanced tools that can accurately analyze gene expression, protein expression and cell specific behaviors. For this, new technologies such as single cell sequencing and transcriptomic techniques are being increasingly used to understand how ncRNAs work in different cell types and during different infection stages.

To report the potential of ncRNAs, we must balance enthusiasm with realism. Many ncRNA types remain largely exploratory, while a few, such as miRNAs, have shown promising results. Our review has largely focused on ncRNAs that have substantial experimental validation. The overall success of using ncRNAs as diagnostic and therapeutic agents requires advances in delivery chemistry, omics tools and careful clinical validation. With careful and coordinated efforts, perhaps the next decade will see the ncRNA based tools move from research laboratories to real-world clinical applications.

## References

[B1] Abe N. Kodama A. Abe H. (2018). “ Preparation of Circular RNA *In Vitro*,” in Circular RNAs: Methods and Protocols.. Eds. Dieterich C. Papantonis A. ( Springer, New York, NY), 181–192. doi: 10.1007/978-1-4939-7562-4_15, PMID:

[B2] Aghajani Mir M. (2024). Illuminating the pathogenic role of SARS-CoV-2: Insights into competing endogenous RNAs (ceRNAs) regulatory networks. Infect. Genet. Evol. J. Mol. Epidemiol. Evol. Genet. Infect. Dis. 122, 105613. doi: 10.1016/j.meegid.2024.105613, PMID: 38844190

[B3] Ahluwalia J. K. Khan S. Z. Soni K. Rawat P. Gupta A. Hariharan M. . (2008). Human cellular microRNA hsa-miR-29a interferes with viral nef protein expression and HIV-1 replication. Retrovirology. 5, 117. doi: 10.1186/1742-4690-5-117, PMID: 19102781 PMC2635386

[B4] Ahmed N. Francis M. E. Ahmed N. Kelvin A. A. Pezacki J. P. (2023). microRNA-185 inhibits SARS-coV-2 infection through the modulation of the host’s lipid microenvironment. Viruses. 15, 1921. doi: 10.3390/v15091921, PMID: 37766327 PMC10536008

[B5] Aldweik M. H. Hisham Y. Aldweik M. H. Hisham Y. (2025). Long non-coding RNAs in viral immunity: from regulatory mechanisms to therapeutic potential. J. Inflamm. Infect. Med. 1, 3. doi: 10.53941/jiim.2025.100015

[B6] Almulhim M. Ghasemian A. Memariani M. Karami F. Yassen A. S. A. Alexiou A. . (2025). Drug repositioning as a promising approach for the eradication of emerging and re-emerging viral agents. Mol. Divers. 29, 5465–5485. doi: 10.1007/s11030-025-11131-8, PMID: 40100484 PMC12638364

[B7] Amaya L. Grigoryan L. Li Z. Lee A. Wender P. A. Pulendran B. . (2023). Circular RNA vaccine induces potent T cell responses. Proc. Natl. Acad. Sci. 120, e2302191120. doi: 10.1073/pnas.2302191120, PMID: 37155869 PMC10193964

[B8] Arroyo M. Salka K. Chorvinsky E. Xuchen X. Abutaleb K. Perez G. F. . (2020). Airway mir-155 responses are associated with TH1 cytokine polarization in young children with viral respiratory infections. PloS One. 15, e0233352. doi: 10.1371/journal.pone.0233352, PMID: 32442188 PMC7244143

[B9] Arslan S. Bakir M. Bayyurt B. Aydemir E. I. Kinaci K. Engin A. (2022). Long noncoding RNA expression analysis in Crimean Congo hemorrhagic fever patients. J. Med. Virol. 94, 3257–3262. doi: 10.1002/jmv.27721, PMID: 35285033

[B10] Aznaourova M. Schmerer N. Janga H. Zhang Z. Pauck K. Bushe J. . (2022). Single-cell RNA sequencing uncovers the nuclear decoy lincRNA PIRAT as a regulator of systemic monocyte immunity during COVID-19. Proc. Natl. Acad. Sci. U. S. A. 119, e2120680119. doi: 10.1073/pnas.2120680119, PMID: 35998224 PMC9457492

[B11] Bahojb Mahdavi S. Z. Jebelli A. Aghbash P. S. Baradaran B. Amini M. Oroojalian F. . (2025). A comprehensive overview on the crosstalk between microRNAs and viral pathogenesis and infection. Med. Res. Rev. 45, 349–425. doi: 10.1002/med.22073, PMID: 39185567 PMC11796338

[B12] Balasubramaniam M. Pandhare J. Dash C. (2018). Are microRNAs important players in HIV-1 infection? An update. Viruses. 10, 110. doi: 10.3390/v10030110, PMID: 29510515 PMC5869503

[B13] Bannazadeh Baghi H. Bayat M. Mehrasa P. Alavi S. M. A. Lotfalizadeh M. H. Memar M. Y. . (2024). Regulatory role of microRNAs in virus-mediated inflammation. J. Inflamm. Lond. Engl. 21, 43. doi: 10.1186/s12950-024-00417-7, PMID: 39497125 PMC11536602

[B14] Barbagallo D. Palermo C. I. Barbagallo C. Battaglia R. Caponnetto A. Spina V. . (2022). Competing endogenous RNA network mediated by circ_3205 in SARS-CoV-2 infected cells. Cell. Mol. Life Sci. 79, 75. doi: 10.1007/s00018-021-04119-8, PMID: 35039944 PMC8763136

[B15] Bartoszewski R. Dabrowski M. Jakiela B. Matalon S. Harrod K. S. Sanak M. . (2020). SARS-CoV-2 may regulate cellular responses through depletion of specific host miRNAs. Am. J. Physiol.-Lung. Cell. Mol. Physiol. 319, L444–L455. doi: 10.1152/ajplung.00252.2020, PMID: 32755307 PMC7473886

[B16] Belousova E. A. Filipenko M. L. Kushlinskii N. E. (2018). Circular RNA: new regulatory molecules. Bull. Exp. Biol. Med. 164, 803–815. doi: 10.1007/s10517-018-4084-z, PMID: 29658072

[B17] Brillante S. Volpe M. Indrieri A. (2024). Advances in microRNA therapeutics: from preclinical to clinical studies. Hum. Gene Ther. 35, 628–648. doi: 10.1089/hum.2024.113, PMID: 39150011

[B18] Caba L. Florea L. Gug C. Dimitriu D. C. Gorduza E. V. (2021). Circular RNA—Is the circle perfect? Biomolecules. 11, 1755. doi: 10.3390/biom11121755, PMID: 34944400 PMC8698871

[B19] Caetano B. F. R. Rocha V. L. Rossini B. C. Dos Santos L. D. Elgui De Oliveira D. (2024). Epstein-Barr Virus miR-BARTs 7 and 9 modulate viral cycle, cell proliferation, and proteomic profiles in Burkitt lymphoma. Tum. Virus Res. 17, 200276. doi: 10.1016/j.tvr.2023.200276, PMID: 38159643 PMC11000110

[B20] Cao Y. Chin A. W. H. Gu H. Li M. Gu Y. Lau S. P. N. . (2025). An interferon-stimulated long non-coding RNA USP30-AS1 as an immune modulator in influenza A virus infection. PloS Pathog. 21, e1012854. doi: 10.1371/journal.ppat.1012854, PMID: 39777915 PMC11750089

[B21] Cao Y. Wu J. Hu Y. Chai Y. Song J. Duan J. . (2023). Virus-induced lncRNA-BTX allows viral replication by regulating intracellular translocation of DHX9 and ILF3 to induce innate escape. Cell Rep. 42, 113262. doi: 10.1016/j.celrep.2023.113262, PMID: 37864796

[B22] Cardon T. Fournier I. Salzet M. (2021). Unveiling a ghost proteome in the glioblastoma non-coding RNAs. Front. Cell Dev. Biol. 9. doi: 10.3389/fcell.2021.703583, PMID: 35004666 PMC8733697

[B23] Carvalho-Silva A. C. Da Silva Junior A. R. Rigaud V. O.-C. Martins W. K. Coelho V. Pfrimer I. A. H. . (2022). A major downregulation of circulating microRNAs in zika acutely infected patients: potential implications in innate and adaptive immune response signaling pathways. Front. Genet. 13. doi: 10.3389/fgene.2022.857728, PMID: 35719399 PMC9199004

[B24] Castillo J. A. Castrillón J. C. Diosa-Toro M. Betancur J. G. St Laurent G. Smit J. M. . (2016). Complex interaction between dengue virus replication and expression of miRNA-133a. BMC Infect. Dis. 16, 29. doi: 10.1186/s12879-016-1364-y, PMID: 26818704 PMC4728791

[B25] Castrillón-Betancur J. C. Urcuqui-Inchima S. (2017). Overexpression of miR-484 and miR-744 in Vero cells alters Dengue virus replication. Mem. Inst. Osw. Cr. 112, 281–291. doi: 10.1590/0074-02760160404, PMID: 28327787 PMC5354610

[B26] Chao T.-C. Zhang Q. Li Z. Tiwari S. K. Qin Y. Yau E. . (2019). The long noncoding RNA HEAL regulates HIV-1 replication through epigenetic regulation of the HIV-1 promoter. mBio. 10, e02016–e02019. doi: 10.1128/mBio.02016-19, PMID: 31551335 PMC6759764

[B27] Chen B. Guo G. Wang G. Zhu Q. Wang L. Shi W. . (2024). ATG7/GAPLINC/IRF3 axis plays a critical role in regulating pathogenesis of influenza A virus. PloS Pathog. 20, e1011958. doi: 10.1371/journal.ppat.1011958, PMID: 38227600 PMC10817227

[B28] Chen D. Ji Q. Liu J. Cheng F. Zheng J. Ma Y. . (2023). MicroRNAs in the regulation of RIG-I-like receptor signaling pathway: possible strategy for viral infection and cancer. Biomolecules. 13, 1344. doi: 10.3390/biom13091344, PMID: 37759744 PMC10526236

[B29] Chen M. Hu J. Zhou X. Gao M. Li N. Yang G. . (2025). Long non-coding RNA THRIL promotes influenza virus replication by inhibiting the antiviral innate immune response. Viruses. 17, 153. doi: 10.3390/v17020153, PMID: 40006907 PMC11861671

[B30] Chen Y. G. Kim M. V. Chen X. Batista P. J. Aoyama S. Wilusz J. E. . (2017). Sensing self and foreign circular RNAs by intron identity. Mol. Cell. 67, 228–238.e5. doi: 10.1016/j.molcel.2017.05.022, PMID: 28625551 PMC5610545

[B31] Chen Y. Shen A. Rider P. J. Yu Y. Wu K. Mu Y. . (2011). A liver-specific microRNA binds to a highly conserved RNA sequence of hepatitis B virus and negatively regulates viral gene expression and replication. FASEB J. 25, 4511–4521. doi: 10.1096/fj.11-187781, PMID: 21903935 PMC3236624

[B32] Chen W.-C. Wei C.-K. Lee J.-C. (2019). MicroRNA-let-7c suppresses hepatitis C virus replication by targeting Bach1 for induction of haem oxygenase-1 expression. J. Viral Hepat. 26, 655–665. doi: 10.1111/jvh.13072, PMID: 30706605

[B33] Chen R.-F. Yang K. D. Lee I.-K. Liu J.-W. Huang C.-H. Lin C.-Y. . (2014). Augmented miR-150 expression associated with depressed SOCS1 expression involved in dengue haemorrhagic fever. J. Infect. 69, 366–374. doi: 10.1016/j.jinf.2014.05.013, PMID: 24907421

[B34] Cheng J. Zhou X. Feng W. Jia M. Zhang X. An T. . (2021). Risk stratification by long non-coding RNAs profiling in COVID-19 patients. J. Cell. Mol. Med. 25, 4753–4764. doi: 10.1111/jcmm.16444, PMID: 33759345 PMC8107096

[B35] Choi J. E. Hur W. Kim J.-H. Li T. Z. Lee E. B. Lee S. W. . (2014). MicroRNA-27a modulates HCV infection in differentiated hepatocyte-like cells from adipose tissue-derived mesenchymal stem cells. PloS One. 9, e91958. doi: 10.1371/journal.pone.0091958, PMID: 24824429 PMC4019502

[B36] Dai X. Zhang W. Zhang H. Sun S. Yu H. Guo Y. . (2014). Modulation of HBV replication by microRNA-15b through targeting hepatocyte nuclear factor 1α. Nucleic Acids Res. 42, 6578–6590. doi: 10.1093/nar/gku260, PMID: 24705650 PMC4041434

[B37] Dass D. Dhotre K. Chakraborty M. Nath A. Banerjee A. Bagchi P. . (2023). miRNAs in herpesvirus infection: powerful regulators in small packages. Viruses. 15, 429. doi: 10.3390/v15020429, PMID: 36851643 PMC9965283

[B38] Filip R. Evers P. Uguccioni S. M. Alonzi R. C. Ahmed N. Ahmed N. . (2025). microRNA-4488 is differentially regulated during dengue virus infection and clearance of the virus. Front. RNA Res. 3. doi: 10.3389/frnar.2025.1566829

[B39] Firoozi Z. Mohammadisoleimani E. Shahi A. Naghizadeh M. M. Mirzaei E. Asad A. G. . (2022). Hsa_circ_0000479/hsa-miR-149-5p/RIG-I, IL-6 axis: A potential novel pathway to regulate immune response against COVID-19. Can. J. Infect. Dis. Med. Microbiol. J. Can. Mal. Infect. Microbiol. Med. 2022, 2762582. doi: 10.1155/2022/2762582, PMID: 36081604 PMC9448594

[B40] Ghani M. U. Zhao G. Pei D. Ma T. Zhao Y. Qu X. . (2025). Inter-species dynamics of non-coding RNAs: Impact on host immunomodulation and pathogen survival. Dev. Comp. Immunol. 164, 105318. doi: 10.1016/j.dci.2025.105318, PMID: 39809336

[B41] Guo H. Liu H. Mitchelson K. Rao H. Luo M. Xie L. . (2011). MicroRNAs-372/373 promote the expression of hepatitis B virus through the targeting of nuclear factor I/B. Hepatol. Baltim. Md. 54, 808–819. doi: 10.1002/hep.24441, PMID: 21608007

[B42] Han J. J. Song H. A. Pierson S. L. Shen-Gunther J. Xia Q. (2023). Emerging infectious diseases are virulent viruses—Are we prepared? An overview. Microorganisms. 11, 2618. doi: 10.3390/microorganisms11112618, PMID: 38004630 PMC10673331

[B43] Henzinger H. Barth D. A. Klec C. Pichler M. (2020). Non-coding RNAs and SARS-related coronaviruses. Viruses. 12, 1374. doi: 10.3390/v12121374, PMID: 33271762 PMC7761185

[B44] Herzog K. Bandiera S. Pernot S. Fauvelle C. Jühling F. Weiss A. . (2020). Functional microRNA screen uncovers O-linked N-acetylglucosamine transferase as a host factor modulating hepatitis C virus morphogenesis and infectivity. Gut. 69, 380–392. doi: 10.1136/gutjnl-2018-317423, PMID: 31076402 PMC7613422

[B45] Hillman T. (2023). The use of plant-derived exosome-like nanoparticles as a delivery system of CRISPR/Cas9-based therapeutics for editing long non-coding RNAs in cancer colon cells. Front. Oncol. 13. doi: 10.3389/fonc.2023.1194350, PMID: 37388221 PMC10301836

[B46] Hoffman S. A. Maldonado Y. A. (2024). Emerging and re-emerging pediatric viral diseases: a continuing global challenge. Pediatr. Res. 95, 480–487. doi: 10.1038/s41390-023-02878-7, PMID: 37940663 PMC10837080

[B47] Hoffmann T. W. Gilles D. Abderrahmane B. (2012). MicroRNAs and hepatitis C virus: Toward the end of miR-122 supremacy. Virol. J. 9, 109. doi: 10.1186/1743-422X-9-109, PMID: 22691570 PMC3489824

[B48] Hong D. S. Kang Y.-K. Borad M. Sachdev J. Ejadi S. Lim H. Y. . (2020). Phase 1 study of MRX34, a liposomal miR-34a mimic, in patients with advanced solid tumours. Br. J. Cancer. 122, 1630–1637. doi: 10.1038/s41416-020-0802-1, PMID: 32238921 PMC7251107

[B49] Hou W. Tian Q. Zheng J. Bonkovsky H. L. (2010). MicroRNA-196 represses bach1 protein and HCV gene expression in human hepatoma cells expressing hepatitis C viral proteins. Hepatol. Baltim. Md. 51, 1494–1504. doi: 10.1002/hep.23401, PMID: 20127796 PMC2862129

[B50] Hu Y. Yu Y. Yang R. Wang R. Pu D. Wang Y. . (2023). The neuropathological mechanism of EV-A71 infection attributes to inflammatory pryoptosis and viral replication via activating the hsa_circ_0045431/hsa_miR_584/NLRP3 regulatory axis. Virus Res. 335, 199195. doi: 10.1016/j.virusres.2023.199195, PMID: 37579846 PMC10450994

[B51] Huang J. Guo Y. Zhao C. Yuan S. Wang Y. Tang G. . (2013). Hepatitis B virus X protein (HBx)-related long noncoding RNA (lncRNA) down-regulated expression by HBx (Dreh) inhibits hepatocellular carcinoma metastasis by targeting the intermediate filament protein vimentin. Hepatol. Baltim. Md. 57, 1882–1892. doi: 10.1002/hep.26195, PMID: 23239537

[B52] Huang J. Wang F. Argyris E. Chen K. Liang Z. Tian H. . (2007). Cellular microRNAs contribute to HIV-1 latency in resting primary CD4+ T lymphocytes. Nat. Med. 13, 1241–1247. doi: 10.1038/nm1639, PMID: 17906637

[B53] Hussein H. A. M. Akula S. M. (2017). miRNA-36 inhibits KSHV, EBV, HSV-2 infection of cells via stifling expression of interferon induced transmembrane protein 1 (IFITM1). Sci. Rep. 7, 17972. doi: 10.1038/s41598-017-18225-w, PMID: 29269892 PMC5740118

[B54] Hwang H. J. Kim Y. K. (2024). Molecular mechanisms of circular RNA translation. Exp. Mol. Med. 56, 1272–1280. doi: 10.1038/s12276-024-01220-3, PMID: 38871818 PMC11263353

[B55] Hynes C. Kakumani P. K. (2024). Regulatory role of RNA-binding proteins in microRNA biogenesis. Front. Mol. Biosci. 11. doi: 10.3389/fmolb.2024.1374843, PMID: 38567098 PMC10985210

[B56] Iizasa H. Kim H. Kartika A. V. Kanehiro Y. Yoshiyama H. (2020). Role of viral and host microRNAs in immune regulation of epstein-barr virus-associated diseases. Front. Immunol. 11. doi: 10.3389/fimmu.2020.00367, PMID: 32194570 PMC7062708

[B57] Ivanov A. Memczak S. Wyler E. Torti F. Porath H. T. Orejuela M. R. . (2015). Analysis of intron sequences reveals hallmarks of circular RNA biogenesis in animals. Cell Rep. 10, 170–177. doi: 10.1016/j.celrep.2014.12.019, PMID: 25558066

[B58] Janssen H. L. A. Reesink H. W. Lawitz E. J. Zeuzem S. Rodriguez-Torres M. Patel K. . (2013). Treatment of HCV infection by targeting microRNA. N. Engl. J. Med. 368, 1685–1694. doi: 10.1056/NEJMoa1209026, PMID: 23534542

[B59] Jiang W. Wang L. Zhang Y. Li H. (2020). Circ-ATP5H induces hepatitis B virus replication and expression by regulating miR-138-5p/TNFAIP3 axis. Cancer Manage. Res. 12, 11031–11040. doi: 10.2147/CMAR.S272983, PMID: 33173336 PMC7648158

[B60] Jin J. Tang S. Xia L. Du R. Xie H. Song J. . (2013). MicroRNA-501 promotes HBV replication by targeting HBXIP. Biochem. Biophys. Res. Commun. 430, 1228–1233. doi: 10.1016/j.bbrc.2012.12.071, PMID: 23266610

[B61] John K. Huntress I. Smith E. Chou H. Tollison T. S. Covarrubias S. . (2025). Human long noncoding RNA VILMIR is induced by major respiratory viral infections and modulates the host interferon response. J. Virol. 99, e0014125. doi: 10.1128/jvi.00141-25, PMID: 40130878 PMC11998520

[B62] Kang J.-G. Majerciak V. Uldrick T. S. Wang X. Kruhlak M. Yarchoan R. . (2011). Kaposi’s sarcoma-associated herpesviral IL-6 and human IL-6 open reading frames contain miRNA binding sites and are subject to cellular miRNA regulation. J. Pathol. 225, 378–389. doi: 10.1002/path.2962, PMID: 21984125 PMC3528401

[B63] Kang L. Xie H. Ye H. Jeyarajan A. J. Warner C. A. Huang Y. . (2023). Hsa_circ_0007321 regulates Zika virus replication through miR-492/NFKBID/NF-κB signaling pathway. J. Virol. 97, e01232–e01223. doi: 10.1128/jvi.01232-23, PMID: 38051045 PMC10734422

[B64] Kazemian P. Yu S.-Y. Thomson S. B. Birkenshaw A. Leavitt B. R. Ross C. J. D. (2022). Lipid-nanoparticle-based delivery of CRISPR/cas9 genome-editing components. Mol. Pharm. 19, 1669–1686. doi: 10.1021/acs.molpharmaceut.1c00916, PMID: 35594500 PMC9176214

[B65] Kristensen L. S. Andersen M. S. Stagsted L. V. W. Ebbesen K. K. Hansen T. B. Kjems J. (2019). The biogenesis, biology and characterization of circular RNAs. Nat. Rev. Genet. 20, 675–691. doi: 10.1038/s41576-019-0158-7, PMID: 31395983

[B66] Kulkarni V. Jayakumar S. Mohan M. Kulkarni S. (2023). Aid or antagonize: nuclear long noncoding RNAs regulate host responses and outcomes of viral infections. Cells. 12, 987. doi: 10.3390/cells12070987, PMID: 37048060 PMC10093752

[B67] Kunden R. D. Khan J. Q. Ghezelbash S. Wilson J. A. (2020). The Role of the Liver-Specific microRNA, miRNA-122 in the HCV Replication Cycle. Int. J. Mol. Sci. 21, 5677. doi: 10.3390/ijms21165677, PMID: 32784807 PMC7460827

[B68] Lai M. Lai R. He B. Wang X. Chen L. Mo Q. (2025). Robust antiviral innate immune response and miRNA regulatory network were identified in ZIKV-infected cells: implications in the pathogenesis of ZIKV infection. Virus Genes. 61, 249–264. doi: 10.1007/s11262-025-02136-4, PMID: 39955676

[B69] Li Y. Ashraf U. Chen Z. Zhou D. Imran M. Ye J. . (2020b). Genome-wide profiling of host-encoded circular RNAs highlights their potential role during the Japanese encephalitis virus-induced neuroinflammatory response. BMC Genomics. 21, 409. doi: 10.1186/s12864-020-06822-5, PMID: 32552669 PMC7301528

[B70] Li J. Chen C. Ma X. Geng G. Liu B. Zhang Y. . (2016). Long noncoding RNA NRON contributes to HIV-1 latency by specifically inducing tat protein degradation. Nat. Commun. 7, 11730. doi: 10.1038/ncomms11730, PMID: 27291871 PMC4909936

[B71] Li S. Duan X. Li Y. Liu B. McGilvray I. Chen L. (2014). MicroRNA-130a inhibits HCV replication by restoring the innate immune response. J. Viral Hepat. 21, 121–128. doi: 10.1111/jvh.12131, PMID: 24383925

[B72] Li J. Li M. Wang X. Sun M. Ma C. Liang W. . (2020a). Long noncoding RNA NRAV promotes respiratory syncytial virus replication by targeting the microRNA miR-509-3p/rab5c axis to regulate vesicle transportation. J. Virol. 94, e00113–e00120. doi: 10.1128/JVI.00113-20, PMID: 32102886 PMC7199404

[B73] Li H.-C. Yang C.-H. Lo S.-Y. (2022). Roles of microRNAs in hepatitis C virus replication and pathogenesis. Viruses. 14, 1776. doi: 10.3390/v14081776, PMID: 36016398 PMC9413378

[B74] Li Y. Zhang C. Qin L. Li D. Zhou G. Dang D. . (2018). Characterization of critical functions of long non-coding RNAs and mRNAs in rhabdomyosarcoma cells and mouse skeletal muscle infected by enterovirus 71 using RNA-seq. Viruses. 10, 556. doi: 10.3390/v10100556, PMID: 30314355 PMC6213062

[B75] Liao Y. Guo S. Liu G. Qiu Z. Wang J. Yang D. . (2021b). Host non-coding RNA regulates influenza A virus replication. Viruses. 14, 51. doi: 10.3390/v14010051, PMID: 35062254 PMC8779696

[B76] Liao R. Liu L. Zhou J. Wei X. Huang P. (2021a). Current molecular biology and therapeutic strategy status and prospects for circRNAs in HBV-associated hepatocellular carcinoma. Front. Oncol. 11. doi: 10.3389/fonc.2021.697747, PMID: 34277444 PMC8284075

[B77] Liu C.-X. Chen L.-L. (2022). Circular RNAs: Characterization, cellular roles, and applications. Cell. 185, 2016–2034. doi: 10.1016/j.cell.2022.04.021, PMID: 35584701

[B78] Liu X. Duan X. Holmes J. A. Li W. Lee S. H. Tu Z. . (2019b). A long noncoding RNA regulates hepatitis C virus infection through interferon alpha-inducible protein 6. Hepatol. Baltim. Md. 69, 1004–1019. doi: 10.1002/hep.30266, PMID: 30199576 PMC6393205

[B79] Liu S. Liu X. Li J. Zhou H. Carr M. J. Zhang Z. . (2019a). Long noncoding RNAs: Novel regulators of virus-host interactions. Rev. Med. Virol. 29, e2046. doi: 10.1002/rmv.2046, PMID: 31016795 PMC7169114

[B80] Liu X. Xiong W. Ye M. Lu T. Yuan K. Chang S. . (2023). Non-coding RNAs expression in SARS-CoV-2 infection: Pathogenesis, clinical significance and therapeutic targets. Signal Transduction Targeting Ther. 8, 441. doi: 10.1038/s41392-023-01669-0, PMID: 38057315 PMC10700414

[B81] Liu H. Yu L. Majerciak V. Meyer T. J. Yi M. Johnson P. F. . (2025). The long noncoding RNA lnc-FANCI-2 intrinsically restricts RAS signaling in human papillomavirus type 16-infected cervical cancer cells. eLife. 13, RP102681. doi: 10.7554/eLife.102681, PMID: 40878909 PMC12396819

[B82] Lu S. Zhu N. Guo W. Wang X. Li K. Yan J. . (2020). RNA-seq revealed a circular RNA-microRNA-mRNA regulatory network in hantaan virus infection. Front. Cell. Infect. Microbiol. 10. doi: 10.3389/fcimb.2020.00097, PMID: 32232013 PMC7083127

[B83] Maarouf M. Wang L. Wang Y. Rai K. R. Chen Y. Fang M. . (2023). Functional involvement of circRNAs in the innate immune responses to viral infection. Viruses. 15, 1697. doi: 10.3390/v15081697, PMID: 37632040 PMC10458642

[B84] Maass P. G. Glažar P. Memczak S. Dittmar G. Hollfinger I. Schreyer L. . (2017). A map of human circular RNAs in clinically relevant tissues. J. Mol. Med. Berl. Ger. 95, 1179–1189. doi: 10.1007/s00109-017-1582-9, PMID: 28842720 PMC5660143

[B85] Mahmoudian-Sani M. R. Asgharzade S. Alghasi A. Saeedi-Boroujeni A. Adnani Sadati S. J. Moradi M. T. (2019). MicroRNA-122 in patients with hepatitis B and hepatitis B virus-associated hepatocellular carcinoma. J. Gastrointest. Oncol. 10, 789–796. doi: 10.21037/jgo.2019.02.14, PMID: 31392060 PMC6657334

[B86] Marchese F. P. Raimondi I. Huarte M. (2017). The multidimensional mechanisms of long noncoding RNA function. Genome Biol. 18, 206. doi: 10.1186/s13059-017-1348-2, PMID: 29084573 PMC5663108

[B87] Matarese A. Gambardella J. Sardu C. Santulli G. (2020). miR-98 regulates TMPRSS2 expression in human endothelial cells: key implications for COVID-19. Biomedicines. 8, 462. doi: 10.3390/biomedicines8110462, PMID: 33143053 PMC7693865

[B88] Mattick J. S. Amaral P. P. Carninci P. Carpenter S. Chang H. Y. Chen L.-L. . (2023). Long non-coding RNAs: definitions, functions, challenges and recommendations. Nat. Rev. Mol. Cell Biol. 24, 430–447. doi: 10.1038/s41580-022-00566-8, PMID: 36596869 PMC10213152

[B89] Media T. S. Cano-Aroca L. Tagawa T. (2025). Non-coding RNAs and immune evasion in human gamma-herpesviruses. Viruses. 17, 1006. doi: 10.3390/v17071006, PMID: 40733622 PMC12298795

[B90] Min J. Li Y. Li X. Wang M. Li H. Bi Y. . (2023). The circRNA circVAMP3 restricts influenza A virus replication by interfering with NP and NS1 proteins. PloS Pathog. 19, e1011577. doi: 10.1371/journal.ppat.1011577, PMID: 37603540 PMC10441791

[B91] Min J. Liu W. Li J. (2022). Emerging role of interferon-induced noncoding RNA in innate antiviral immunity. Viruses. 14, 2607. doi: 10.3390/v14122607, PMID: 36560611 PMC9780829

[B92] Moazzam-Jazi M. Lanjanian H. Maleknia S. Hedayati M. Daneshpour M. S. (2021). Interplay between SARS-CoV-2 and human long non-coding RNAs. J. Cell. Mol. Med. 25, 5823–5827. doi: 10.1111/jcmm.16596, PMID: 33969601 PMC8184717

[B93] Moles R. Bellon M. Nicot C. (2015). STAT1: A Novel Target of miR-150 and miR-223 Is Involved in the Proliferation of HTLV-I–Transformed and ATL Cells. Neoplasia. N. Y. N. 17, 449–462. doi: 10.1016/j.neo.2015.04.005, PMID: 26025667 PMC4468372

[B94] Mukherjee A. Shrivastava S. Bhanja Chowdhury J. Ray R. Ray R. B. (2014). Transcriptional suppression of miR-181c by hepatitis C virus enhances homeobox A1 expression. J. Virol. 88, 7929–7940. doi: 10.1128/JVI.00787-14, PMID: 24789793 PMC4097774

[B95] Nahand J. S. Karimzadeh M. R. Nezamnia M. Fatemipour M. Khatami A. Jamshidi S. . (2020). The role of miR-146a in viral infection. IUBMB Life. 72, 343–360. doi: 10.1002/iub.2222, PMID: 31889417

[B96] Nishitsuji H. Ujino S. Yoshio S. Sugiyama M. Mizokami M. Kanto T. . (2016). Long noncoding RNA 32 contributes to antiviral responses by controlling interferon-stimulated gene expression. Proc. Natl. Acad. Sci. 113, 10388–10393. doi: 10.1073/pnas.1525022113, PMID: 27582466 PMC5027408

[B97] Nojima T. Proudfoot N. J. (2022). Mechanisms of lncRNA biogenesis as revealed by nascent transcriptomics. Nat. Rev. Mol. Cell Biol. 23, 389–406. doi: 10.1038/s41580-021-00447-6, PMID: 35079163

[B98] O’Brien J. Hayder H. Zayed Y. Peng C. (2018). Overview of microRNA biogenesis, mechanisms of actions, and circulation. Front. Endocrinol. 9. doi: 10.3389/fendo.2018.00402, PMID: 30123182 PMC6085463

[B99] Oo J. A. Brandes R. P. Leisegang M. S. (2022). Long non-coding RNAs: novel regulators of cellular physiology and function. Pflugers. Arch. 474, 191–204. doi: 10.1007/s00424-021-02641-z, PMID: 34791525 PMC8766390

[B100] Ouyang J. Zhu X. Chen Y. Wei H. Chen Q. Chi X. . (2014). NRAV, a long noncoding RNA, modulates antiviral responses through suppression of interferon-stimulated gene transcription. Cell Host Microbe. 16, 616–626. doi: 10.1016/j.chom.2014.10.001, PMID: 25525793 PMC7104942

[B101] Panda A. C. (2018). Circular RNAs act as miRNA sponges. Adv. Exp. Med. Biol. 1087, 67–79. doi: 10.1007/978-981-13-1426-1_6, PMID: 30259358

[B102] Pandey A. D. Goswami S. Shukla S. Das S. Ghosal S. Pal M. . (2017). Correlation of altered expression of a long non-coding RNA, NEAT1, in peripheral blood mononuclear cells with dengue disease progression. J. Infect. 75, 541–554. doi: 10.1016/j.jinf.2017.09.016, PMID: 29031635

[B103] Panigrahi M. Palmer M. A. Wilson J. A. (2022). MicroRNA-122 regulation of HCV infections: insights from studies of miR-122-independent replication. Pathogens. 11, 1005. doi: 10.3390/pathogens11091005, PMID: 36145436 PMC9504723

[B104] Patrycy M. Chodkowski M. Krzyzowska M. (2022). Role of microglia in herpesvirus-related neuroinflammation and neurodegeneration. Pathogens. 11, 809. doi: 10.3390/pathogens11070809, PMID: 35890053 PMC9324537

[B105] Peng S. Wang J. Wei S. Li C. Zhou K. Hu J. . (2018). Endogenous cellular microRNAs mediate antiviral defense against influenza A virus. Mol. Ther. Nucleic Acids. 10, 361–375. doi: 10.1016/j.omtn.2017.12.016, PMID: 29499948 PMC5862538

[B106] Pius-Sadowska E. Kulig P. Niedźwiedź A. Baumert B. Rogińska D. Łuczkowska K. . (2025). The micro-RNA expression profile predicts the severity of SARS-CoV-2 infection. Sci. Rep. 15, 17139. doi: 10.1038/s41598-025-01229-2, PMID: 40382351 PMC12085561

[B107] Qiu H. Yang B. Chen Y. Zhu Q. Wen F. Peng M. . (2023). Influenza A Virus-Induced circRNA circMerTK Negatively Regulates Innate Antiviral Responses. Microbiol. Spectr. 11, e03637–e03622. doi: 10.1128/spectrum.03637-22, PMID: 36847523 PMC10100971

[B108] Qu D. Sun W.-W. Li L. Ma L. Sun L. Jin X. . (2019). Long noncoding RNA MALAT1 releases epigenetic silencing of HIV-1 replication by displacing the polycomb repressive complex 2 from binding to the LTR promoter. Nucleic Acids Res. 47, 3013–3027. doi: 10.1093/nar/gkz117, PMID: 30788509 PMC6451131

[B109] Qu L. Yi Z. Shen Y. Lin L. Chen F. Xu Y. . (2022). Circular RNA vaccines against SARS-CoV-2 and emerging variants. Cell. 185, 1728–1744.e16. doi: 10.1016/j.cell.2022.03.044, PMID: 35460644 PMC8971115

[B110] Rahni Z. Hosseini S. M. Shahrokh S. Saeedi Niasar M. Shoraka S. Mirjalali H. . (2023). Long non-coding RNAs ANRIL, THRIL, and NEAT1 as potential circulating biomarkers of SARS-CoV-2 infection and disease severity. Virus Res. 336, 199214. doi: 10.1016/j.virusres.2023.199214, PMID: 37657511 PMC10502354

[B111] Rai K. R. Liao Y. Cai M. Qiu H. Wen F. Peng M. . (2022). MIR155HG plays a bivalent role in regulating innate antiviral immunity by encoding long noncoding RNA-155 and microRNA-155-5p. mBio. 13, e0251022. doi: 10.1128/mbio.02510-22, PMID: 36321836 PMC9765511

[B112] Rizkita L. D. Astuti I. (2021). The potential of miRNA-based therapeutics in severe acute respiratory syndrome coronavirus 2 (SARS-CoV-2) infection: A review. J. Pharm. Anal. 11, 265–271. doi: 10.1016/j.jpha.2021.03.003, PMID: 33782640 PMC7989072

[B113] Rodrigues A. C. Adamoski D. Genelhould G. Zhen F. Yamaguto G. E. Araujo-Souza P. S. . (2021). NEAT1 and MALAT1 are highly expressed in saliva and nasopharyngeal swab samples of COVID-19 patients. Mol. Oral. Microbiol. 36, 291–294. doi: 10.1111/omi.12351, PMID: 34463043 PMC8661855

[B114] Rossetto C. C. Pari G. S. (2014). PAN’s labyrinth: molecular biology of kaposi’s sarcoma-associated herpesvirus (KSHV) PAN RNA, a multifunctional long noncoding RNA. Viruses. 6, 4212–4226. doi: 10.3390/v6114212, PMID: 25375885 PMC4246217

[B115] Santer L. Bär C. Thum T. (2019). Circular RNAs: A novel class of functional RNA molecules with a therapeutic perspective. Mol. Ther. 27, 1350–1363. doi: 10.1016/j.ymthe.2019.07.001, PMID: 31324392 PMC6697450

[B116] Schwartz D. A. (2021). Prioritizing the continuing global challenges to emerging and reemerging viral infections. Front. Virol. 1. doi: 10.3389/fviro.2021.701054

[B117] Shirahama S. Onoguchi-Mizutani R. Kawata K. TaNiue K. Miki A. Kato A. . (2020). Long noncoding RNA U90926 is crucial for herpes simplex virus type 1 proliferation in murine retinal photoreceptor cells. Sci. Rep. 10, 19406. doi: 10.1038/s41598-020-76450-2, PMID: 33173149 PMC7656448

[B118] Song L. Liu H. Gao S. Jiang W. Huang W. (2010). Cellular microRNAs inhibit replication of the H1N1 influenza A virus in infected cells. J. Virol. 84, 8849–8860. doi: 10.1128/JVI.00456-10, PMID: 20554777 PMC2919005

[B119] Statello L. Guo C.-J. Chen L.-L. Huarte M. (2021). Gene regulation by long non-coding RNAs and its biological functions. Nat. Rev. Mol. Cell Biol. 22, 96–118. doi: 10.1038/s41580-020-00315-9, PMID: 33353982 PMC7754182

[B120] Steiner D. F. Thomas M. F. Hu J. K. Yang Z. Babiarz J. E. Allen C. D. C. . (2011). MicroRNA-29 regulates T-box transcription factors and interferon-γ Production in helper T cells. Immunity. 35, 169–181. doi: 10.1016/j.immuni.2011.07.009, PMID: 21820330 PMC3361370

[B121] Suarez B. Prats-Mari L. Unfried J. P. Fortes P. (2020). LncRNAs in the type I interferon antiviral response. Int. J. Mol. Sci. 21, 6447. doi: 10.3390/ijms21176447, PMID: 32899429 PMC7503479

[B122] Szczesniak I. Baliga-Gil A. Jarmolowicz A. Soszynska-Jozwiak M. Kierzek E. (2023). Structural and functional RNA motifs of SARS-coV-2 and influenza A virus as a target of viral inhibitors. Int. J. Mol. Sci. 24, 1232. doi: 10.3390/ijms24021232, PMID: 36674746 PMC9860923

[B123] Tagawa T. Oh D. Dremel S. Mahesh G. Koparde V. N. Duncan G. . (2023). A virus-induced circular RNA maintains latent infection of Kaposi’s sarcoma herpesvirus. Proc. Natl. Acad. Sci. 120, e2212864120. doi: 10.1073/pnas.2212864120, PMID: 36724259 PMC9963958

[B124] Tycowski K. T. Guo Y. E. Lee N. Moss W. N. Vallery T. K. Xie M. . (2015). Viral noncoding RNAs: more surprises. Genes Dev. 29, 567–584. doi: 10.1101/gad.259077.115, PMID: 25792595 PMC4378190

[B125] Ullah A. Yu X. Odenthal M. Meemboor S. Ahmad B. Rehman I. U. . (2022). Circulating microRNA-122 in HCV cirrhotic patients with high frequency of genotype 3. PloS One. 17, e0268526. doi: 10.1371/journal.pone.0268526, PMID: 35617369 PMC9135289

[B126] Ungerleider N. Bullard W. Kara M. Wang X. Roberts C. Renne R. . (2021). EBV miRNAs are potent effectors of tumor cell transcriptome remodeling in promoting immune escape. PloS Pathog. 17, e1009217. doi: 10.1371/journal.ppat.1009217, PMID: 33956915 PMC8130916

[B127] Vaddadi K. Gandikota C. Huang C. Liang Y. Liu L. (2023). Cellular microRNAs target SARS-CoV-2 spike protein and restrict viral replication. Am. J. Physiol. Cell Physiol. 325, C420–C428. doi: 10.1152/ajpcell.00516.2022, PMID: 37399496 PMC10390048

[B128] Van Solingen C. Cyr Y. Scacalossi K. R. De Vries M. Barrett T. J. De Jong A. . (2022). Long noncoding RNA CHROMR regulates antiviral immunity in humans. Proc. Natl. Acad. Sci. 119, e2210321119. doi: 10.1073/pnas.2210321119, PMID: 36001732 PMC9477407

[B129] Venkatesan A. Barik A. Paul D. Muthaiyan M. Das R. (2022). Identification of novel lncRNA by reanalysis of RNA-seq data in Zika Virus Infected hiNPCs. VirusDisease. 33, 185–193. doi: 10.1007/s13337-022-00771-1, PMID: 35991697 PMC9381673

[B130] Verhoeven R. J. A. Tong S. Mok B. W.-Y. Liu J. He S. Zong J. . (2019). Epstein-barr virus BART long non-coding RNAs function as epigenetic modulators in nasopharyngeal carcinoma. Front. Oncol. 9. doi: 10.3389/fonc.2019.01120, PMID: 31696060 PMC6817499

[B131] Wang P. (2018). The opening of pandora’s box: an emerging role of long noncoding RNA in viral infections. Front. Immunol. 9. doi: 10.3389/fimmu.2018.03138, PMID: 30740112 PMC6355698

[B132] Wang J. Cen S. (2020). Roles of lncRNAs in influenza virus infection. Emerg. Microbes Infect. 9, 1407–1414. doi: 10.1080/22221751.2020.1778429, PMID: 32543285 PMC7473136

[B133] Wang J. Cheng Y. Wang L. Sun A. Lin Z. Zhu W. . (2022). Chicken miR-126-5p negatively regulates antiviral innate immunity by targeting TRAF3. Vet. Res. 53, 82. doi: 10.1186/s13567-022-01098-x, PMID: 36224663 PMC9559812

[B134] Wang M. Gu B. Yao G. Li P. Wang K. (2020). Circular RNA expression profiles and the pro-tumorigenic function of circRNA_10156 in hepatitis B virus-related liver cancer. Int. J. Med. Sci. 17, 1351–1365. doi: 10.7150/ijms.45637, PMID: 32624692 PMC7330659

[B135] Wang X.-J. Jiang S.-C. Wei H.-X. Deng S.-Q. He C. Peng H.-J. (2017c). The differential expression and possible function of long noncoding RNAs in liver cells infected by dengue virus. Am. J. Trop. Med. Hyg. 97, 1904–1912. doi: 10.4269/ajtmh.17-0307, PMID: 29016307 PMC5805055

[B136] Wang Y. Li Y. (2018). MiR-29c inhibits HCV replication via activation of type I IFN response by targeting STAT3 in JFH-1-infected Huh7 cells. RSC. Adv. 8, 8164–8172. doi: 10.1039/c7ra12815k, PMID: 35542013 PMC9078521

[B137] Wang S. Li X. Liu G. Qiu Z. Wang J. Yang D. . (2024). Advances in the understanding of circRNAs that influence viral replication in host cells. Med. Microbiol. Immunol. (Berl.). 213, 1. doi: 10.1007/s00430-023-00784-7, PMID: 38329596

[B138] Wang P. Xu J. Wang Y. Cao X. (2017b). An interferon-independent lncRNA promotes viral replication by modulating cellular metabolism. Science. 358, 1051–1055. doi: 10.1126/science.aao0409, PMID: 29074580

[B139] Wang M. Yu F. Wu W. Zhang Y. Chang W. Ponnusamy M. . (2017a). Circular RNAs: A novel type of non-coding RNA and their potential implications in antiviral immunity. Int. J. Biol. Sci. 13, 1497–1506. doi: 10.7150/ijbs.22531, PMID: 29230098 PMC5723916

[B140] Wang Q. Zhang D. Feng W. Guo Y. Sun X. Zhang M. . (2022). Long noncoding RNA TSPOAP1 antisense RNA 1 negatively modulates type I IFN signaling to facilitate influenza A virus replication. J. Med. Virol. 94, 557–566. doi: 10.1002/jmv.25483, PMID: 30968963

[B141] Wang J. Zhang Y. Zhu F. Chen L. Wei Y. Zhu Q. . (2021). CircRNA expression profiling and bioinformatics analysis indicate the potential biological role and clinical significance of circRNA in influenza A virus-induced lung injury. J. Biosci. 46, 38. doi: 10.1007/s12038-021-00152-8, PMID: 33969826 PMC8060339

[B142] Wen W. He Z. Jing Q. Hu Y. Lin C. Zhou R. . (2015). Cellular microRNA-miR-548g-3p modulates the replication of dengue virus. J. Infect. 70, 631–640. doi: 10.1016/j.jinf.2014.12.001, PMID: 25499200

[B143] Withers J. B. Mondol V. Pawlica P. Rosa-Mercado N. A. Tycowski K. T. Ghasempur S. . (2019). Idiosyncrasies of viral noncoding RNAs provide insights into host cell biology. Annu. Rev. Virol. 6, 297–317. doi: 10.1146/annurev-virology-092818-015811, PMID: 31039329 PMC6768742

[B144] Wong R. R. Abd-Aziz N. Affendi S. Poh C. L. (2020). Role of microRNAs in antiviral responses to dengue infection. J. Biomed. Sci. 27, 4. doi: 10.1186/s12929-019-0614-x, PMID: 31898495 PMC6941309

[B145] Xiao M. Chen Y. Wang S. Liu S. Rai K. R. Chen B. . (2021). Long noncoding RNA IFITM4P regulates host antiviral responses by acting as a competing endogenous RNA. J. Virol. 95, e0027721. doi: 10.1128/jvi.00277-21, PMID: 34287042 PMC8513474

[B146] Xie Q. Chen S. Tian R. Huang X. Deng R. Xue B. . (2018a). Long noncoding RNA ITPRIP-1 positively regulates the innate immune response through promotion of oligomerization and activation of MDA5. J. Virol. 92, e00507–e00518. doi: 10.1128/JVI.00507-18, PMID: 29899107 PMC6096792

[B147] Xie Y. He S. Wang J. (2018b). MicroRNA-373 facilitates HSV-1 replication through suppression of type I IFN response by targeting IRF1. Biomed. Pharmacother. 97, 1409–1416. doi: 10.1016/j.biopha.2017.11.071, PMID: 29156530

[B148] Yan W. Fu X. Li H. Wang K. Song C. Hou C. . (2024). The long non-coding RNA lncRNA-DRNR enhances infectious bronchitis virus replication by targeting chicken JMJD6 and modulating interferon-stimulated genes expression via the JAK-STAT signalling pathway. Vet. Res. 55, 141. doi: 10.1186/s13567-024-01396-6, PMID: 39501382 PMC11539454

[B149] Yan Q. Ma X. Shen C. Cao X. Feng N. Qin D. . (2014). Inhibition of kaposi’s sarcoma-associated herpesvirus lytic replication by HIV-1 nef and cellular microRNA hsa-miR-1258. J. Virol. 88, 4987–5000. doi: 10.1128/jvi.00025-14, PMID: 24554664 PMC3993842

[B150] Yan H. Zhu X. Zhang D. Zhang K. Shi N. Liu X. (2025). Hsa_circ_0008085 acts as a miR-146a-5p sponge to suppress influenza a virus replication via modulating of TRAF6. Int. Immunopharmacol. 157, 114743. doi: 10.1016/j.intimp.2025.114743, PMID: 40306111

[B151] Yao S. Jia X. Wang F. Sheng L. Song P. Cao Y. . (2021). CircRNA ARFGEF1 functions as a ceRNA to promote oncogenic KSHV-encoded viral interferon regulatory factor induction of cell invasion and angiogenesis by upregulating glutaredoxin 3. PloS Pathog. 17, e1009294. doi: 10.1371/journal.ppat.1009294, PMID: 33539420 PMC7888650

[B152] Yin X. Li H. Zhou Y. (2024). Circular RNAs in viral infection and antiviral treatment. Cells. 13, 2033. doi: 10.3390/cells13232033, PMID: 39682781 PMC11640649

[B153] Yu J. Ding W.-B. Wang M.-C. Guo X.-G. Xu J. Xu Q.-G. . (2020a). Plasma circular RNA panel to diagnose hepatitis B virus-related hepatocellular carcinoma: A large-scale, multicenter study. Int. J. Cancer. 146, 1754–1763. doi: 10.1002/ijc.32647, PMID: 31456215

[B154] Yu X. He Q. Kong Q. (2024). Multidisciplinary approaches to combat emerging viruses: diagnostics, therapeutic gene and vaccine delivery, and nanotherapeutics. Front. Microbiol. 15. doi: 10.3389/fmicb.2024.1387623, PMID: 38966392 PMC11222566

[B155] Yu J. Li W. Hou G. Sun D. Yang Y. Yuan S. . (2023). Circular RNA cFAM210A, degradable by HBx, inhibits HCC tumorigenesis by suppressing YBX1 transactivation. Exp. Mol. Med. 55, 2390–2401. doi: 10.1038/s12276-023-01108-8, PMID: 37907737 PMC10689457

[B156] Yu S. Li N. Wang J. Fu Y. Huang Y. Yi P. . (2020b). Correlation of long noncoding RNA SEMA6A-AS1 expression with clinical outcome in HBV-related hepatocellular carcinoma. Clin. Ther. 42, 439–447. doi: 10.1016/j.clinthera.2020.01.012, PMID: 32070484

[B157] Zhang X. Chu H. Wen L. Shuai H. Yang D. Wang Y. . (2020). Competing endogenous RNA network profiling reveals novel host dependency factors required for MERS-CoV propagation. Emerg. Microbes Infect. 9, 733–746. doi: 10.1080/22221751.2020.1738277, PMID: 32223537 PMC7170352

[B158] Zhang X. Hou J. Lu M. (2013). Regulation of hepatitis B virus replication by epigenetic mechanisms and microRNAs. Front. Genet. 4. doi: 10.3389/fgene.2013.00202, PMID: 24133502 PMC3796260

[B159] Zhang G. Li Y. Zheng S. Liu M. Li X. Tang H. (2010). Suppression of hepatitis B virus replication by microRNA-199a-3p and microRNA-210. Antiviral Res. 88, 169–175. doi: 10.1016/j.antiviral.2010.08.008, PMID: 20728471

[B160] Zhang S. Liu N. Cao P. Qin Q. Li J. Yang L. . (2024). LncRNA BC200 promotes the development of EBV-associated nasopharyngeal carcinoma by competitively binding to miR-6834-5p to upregulate TYMS expression. Int. J. Biol. Macromol. 278, 134837. doi: 10.1016/j.ijbiomac.2024.134837, PMID: 39179085

[B161] Zhang P. Wu W. Chen Q. Chen M. (2019). Non-coding RNAs and their integrated networks. J. Integr. Bioinforma. 16, 20190027. doi: 10.1515/jib-2019-0027, PMID: 31301674 PMC6798851

[B162] Zhao Y. Li H. Du H. Yin Z. He M. Fan J. . (2023). A Kaposi’s sarcoma-associated herpes virus-encoded microRNA contributes to dilated cardiomyopathy. Signal Transduction Targeting Ther. 8, 226. doi: 10.1038/s41392-023-01434-3, PMID: 37291118 PMC10250357

[B163] Zhao L. Xia M. Wang K. Lai C. Fan H. Gu H. . (2020). A long non-coding RNA IVRPIE promotes host antiviral immune responses through regulating interferon β1 and ISG expression. Front. Microbiol. 11. doi: 10.3389/fmicb.2020.00260, PMID: 32153544 PMC7044153

[B164] Zhu B. Ye J. Nie Y. Ashraf U. Zohaib A. Duan X. . (2015). MicroRNA-15b modulates Japanese encephalitis virus–mediated inflammation via targeting RNF125. J. Immunol. 195, 2251–2262. doi: 10.4049/jimmunol.1500370, PMID: 26202983

