# Correction: Non-coding RNAs in the viral host-pathogen interaction: molecular regulation and therapeutic potential

**DOI:** 10.3389/fcimb.2025.1772627

**Published:** 2026-01-06

**Authors:** Maham Yamin, Nirmin Alsahafi, Rwaa Hussin Abdulal, Muhammad Asad, Mohammad Bosaeed, Ali Zohaib

**Affiliations:** 1 Rawalpindi Medical University, Rawalpindi, Pakistan; 2 Infectious Disease Research Department, King Abdullah International Medical Research Center, Jeddah, Saudi Arabia; 3 King Saud bin Abdulaziz University for Health Sciences, Jeddah, Saudi Arabia; 4 Ministry of the National Guard - Health Affairs, Jeddah, Saudi Arabia; 5 Huazhong Agricultural University, Wuhan, China; 6 Infectious Disease Research Department, King Abdullah International Medical Research Center, Riyadh, Saudi Arabia; 7 King Saud bin Abdulaziz University for Health Sciences, Riyadh, Saudi Arabia; 8 Ministry of the National Guard - Health Affairs, Riyadh, Saudi Arabia

**Keywords:** circRNA, host–pathogen interaction, long non-coding RNAs, microRNAs, non-coding RNAs, RNA therapeutics, viral infection

An incorrect **Funding** statement was provided. In the published article, the incorrect **Funding** statement reads as “The author(s) declared that financial support was not received for this work and/or its publication. This work was supported by the King Abdullah International Medical Research Center (NRJ25/049/9)”. The correct **Funding** statement reads:

“The author(s) declared that financial support was received for this work and/or its publication. This work was supported by the King Abdullah International Medical Research Center (NRJ25/049/9)”

A correction has been made to the section Section *6.3 Translational applications of circRNAs*, Paragraph Number 2, First Line. The first three words of the sentence are duplicated and read as “Not only are Not only are circRNAs highly stable, but they also trigger strong immune responses, making them ideal candidates for RNA-based vaccine platforms”.

The correct statement is “Not only are circRNAs highly stable, but they also trigger strong immune responses, making them ideal candidates for RNA-based vaccine platforms.”

The original version of this article has been updated.

